# TNF-α Contributes to Caspase-3 Independent Apoptosis in Neuroblastoma Cells: Role of NFAT

**DOI:** 10.1371/journal.pone.0016100

**Published:** 2011-01-27

**Authors:** Susana Álvarez, Almudena Blanco, Manuel Fresno, Ma Ángeles Muñoz-Fernández

**Affiliations:** 1 Centro de Biología Molecular, Consejo Superior de Investigaciones Científicas, Universidad Autónoma de Madrid, Madrid, Spain; 2 Lab Inmuno-Biología Molecular, Hospital General Universitario Gregorio Marañón, Madrid, Spain; Boston University School of Medicine, United States of America

## Abstract

There is increasing evidence that soluble factors in inflammatory central nervous system diseases not only regulate the inflammatory process but also directly influence electrophysiological membrane properties of neurons and astrocytes. In this context, the cytokine TNF-α (tumor necrosis factor-α) has complex injury promoting, as well as protective, effects on neuronal viability. Up-regulated TNF-α expression has also been found in various neurodegenerative diseases such as cerebral malaria, AIDS dementia, Alzheimer's disease, multiple sclerosis, and stroke, suggesting a potential pathogenic role of TNF-α in these diseases as well. We used the neuroblastoma cells SK-N-MC. Transcriptional activity was measured using luciferase reporter gene assays by using lipofectin. We performed cotransfection experiments of NFAT (nuclear factor of activated T cells) promoter constructed with a dominant negative version of NFAT (dn-NFAT). Cell death was performed by MTT (3-(4,5-dimethylthiazol-2-yl)5,5-diphenyltetrazolium bromide) and TUNEL assays. NFAT translocation was confirmed by Western blot. Involvement of NFAT in cell death was assessed by using VIVIT. P53, Fas-L, caspase-3, and caspase-9 expressions were carried out by Western blot. The mechanisms involved in TNF-α-induced cell death were assessed by using microarray analysis. TNF-α causes neuronal cell death in the absence of glia. TNF-α treatment results in nuclear translocation of NFAT through activation of calcineurin in a Ca^2+^ independent manner. We demonstrated the involvement of FasL/Fas, cytochrome c, and caspase-9 but the lack of caspase-3 activation. NB cell death was absolutely reverted in the presence of VIVIT, and partially diminished by anti-Fas treatment. These data demonstrate that TNF-α promotes FasL expression through NFAT activation in neuroblastoma cells and this event leads to increased apoptosis through independent caspase-3 activation.

## Introduction

Tumor necrosis factor-α (TNF-α), the most widely studied cytokine, plays many roles as a signaling and as an effector molecule in both physiology and pathophysiology of the central nervous system (CNS) [1]. On the one hand, TNF-α plays a critical role in brain development, brain physiology, synaptic plasticity, sleep, circadian rhythm, normal behavior, etc [2]. It has been shown to induce the activation of glial cells and macrophages for the production of a variety of neurotoxins and to initiate the death process in oligodendrocytes and neurons [3]. The pleiotropic actions of TNF-α are mediated through two distinct cell surface receptors: 55 kDa TNFR1 (p55, or CD120a) and 75 kDa TNFR2 (also called p75, and CD120b) (reviewed in [4]). Although it has been described that both TNF-α receptors in the brain are expressed by neurons and glia [5], [6], receptor distribution varies depending upon activation of either apoptosis or inflammatory regulation [7], [8], and although the functions of p75 in the brain are still unclear, activation of p55 initiates signals leading to neuronal apoptosis. These differential patterns of localization of TNF-α receptors in neuronal and glial cells, their state of activation and the down-stream effectors, all are thought to play an important role in determining whether TNF-α will exert a beneficial or harmful effect on CNS. In addition, TNFRs mediate the activation of several transcription factors leading to enhanced gene expression (reviewed in [9]).

In the CNS, resident macrophages, astrocytes and microglia are able to produce TNF-α, which seems to be proinflammatory during the acute phase of CNS inflammatory responses, but immunosuppressive during the chronic phase. One effect through which TNF-α is neurotoxic is by over-stimulation of the glutamate receptors, such as the N-methyl-D-aspartate receptor.

The nuclear factor of activated T cells (NFAT) family of proteins was first discovered by identification of factors involved in the upregulation of IL-2 in response to TCR stimulation [10]. Since that time, NFAT proteins have been implicated in a wide variety of cellular processes including cardiac hypertrophy, learning and memory, and adipocyte differentiation. NFAT transcription factors are highly phosphorylated proteins residing in the cytoplasm of resting cells, and are regulated primarily through calcium levels in the cell. Upon stimulation, an increase in intracellular calcium turns on the serine/threonine phosphatase calcineurin (CaN), which then binds to NFAT and dephosphorylates the protein, causing its nuclear translocation, where they orchestrate developmental and activation programs in diverse cell types. Intense CaN expression localized to activated astrocytes surrounding amyloid plaques in AD model mice [11]. In addition, Aβ_(1-42)_ peptides, the primary constituents of amyloid plaques, have been shown to potently stimulate CaN-dependent signaling in cell culture models, brain slices, and intact animals [11], [12].

Apoptosis is the predominant form of cell death triggered in vivo and in vitro by drugs in hematologic malignancies [13]. There are two major routes by which apoptosis can be induced: (1) the mitochondrial or intrinsic apoptosis pathway; and (2) the death receptor-mediated or extrinsic apoptosis pathway. Conversely, the mitochondrial apoptotic pathway, activated by developmental cues or cytotoxic stimuli is independent of caspase-8 and Fas-associated death domain protein (FADD), but involves mitochondrial release of cytochrome c, which promotes apoptotic protease-activating factor-1 (Apaf-1)-mediated activation of caspase-9 [14]. Following its activation, caspase-9 activates the downstream effector caspase cascade [15]. Initiation of the extrinsic apoptosis pathway involves ligand-induced aggregation of death receptors and activation of procaspase-8 or procaspase-10 within the death-inducing signaling complex [16]. The intrinsic and extrinsic apoptotic pathways converge at the level of caspase-3 activation.

We report that TNF-α causes cell death in human neuroblastoma (NB) cells through NFAT activation and upregulation of FasL protein. In addition, TNF-α-induced cell death involves release of cytochrome c from the mitochondria that leads to caspase-9 activation.

## Results

### TNF-α causes neuronal cell death in the absence of glia

**Figure 1:**
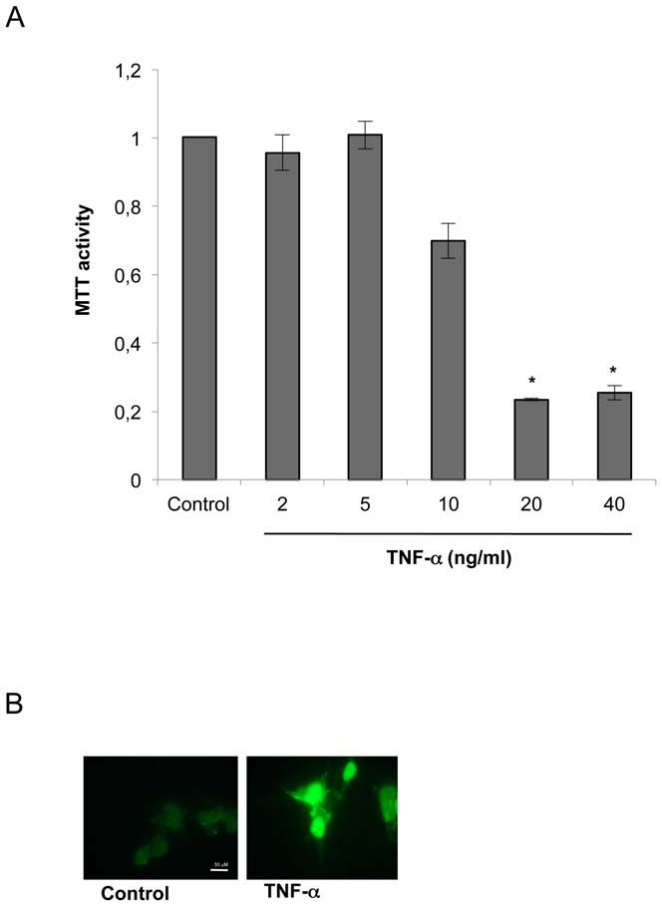
TNF-α causes cell death of NB cells in the absence of glia. A) SK-N-MC cells were treated with TNF-α (2, 5, 10, 20, and 40 ng/ml) and examined using the MTT assay after 24 h. Data are mean ± SD of three independent experiments performed in triplicate. Significant difference from controls: *p<0.01. B) Apoptosis determined by TUNEL. SK-N-MC cells were stained and TUNEL visualized under a fluorescence microscope with either 100 magnification. Bar: 50 µm.

NB cells were exposed to control medium or to medium containing various concentrations of TNF-α, and cell survival was measured by MTT assay 24 h later. TNF-α evoked a dose-dependent decrease in cell survival starting with a concentration of 10 ng/ml (Figure 1A).

We next examined whether cell death induced by TNF-α was apoptotic. SK-N-MC cells were treated with TNF-α for 24 h and assayed for apoptosis by using the TUNEL staining method, which detects apoptosis-associated DNA strand breaks. As shown in Figure 1B, the number of TUNEL-positive cells increased after TNF-α treatment.

### Intracellular signaling associated with TNF-α-mediated neurotoxicity

**Figure 2:**
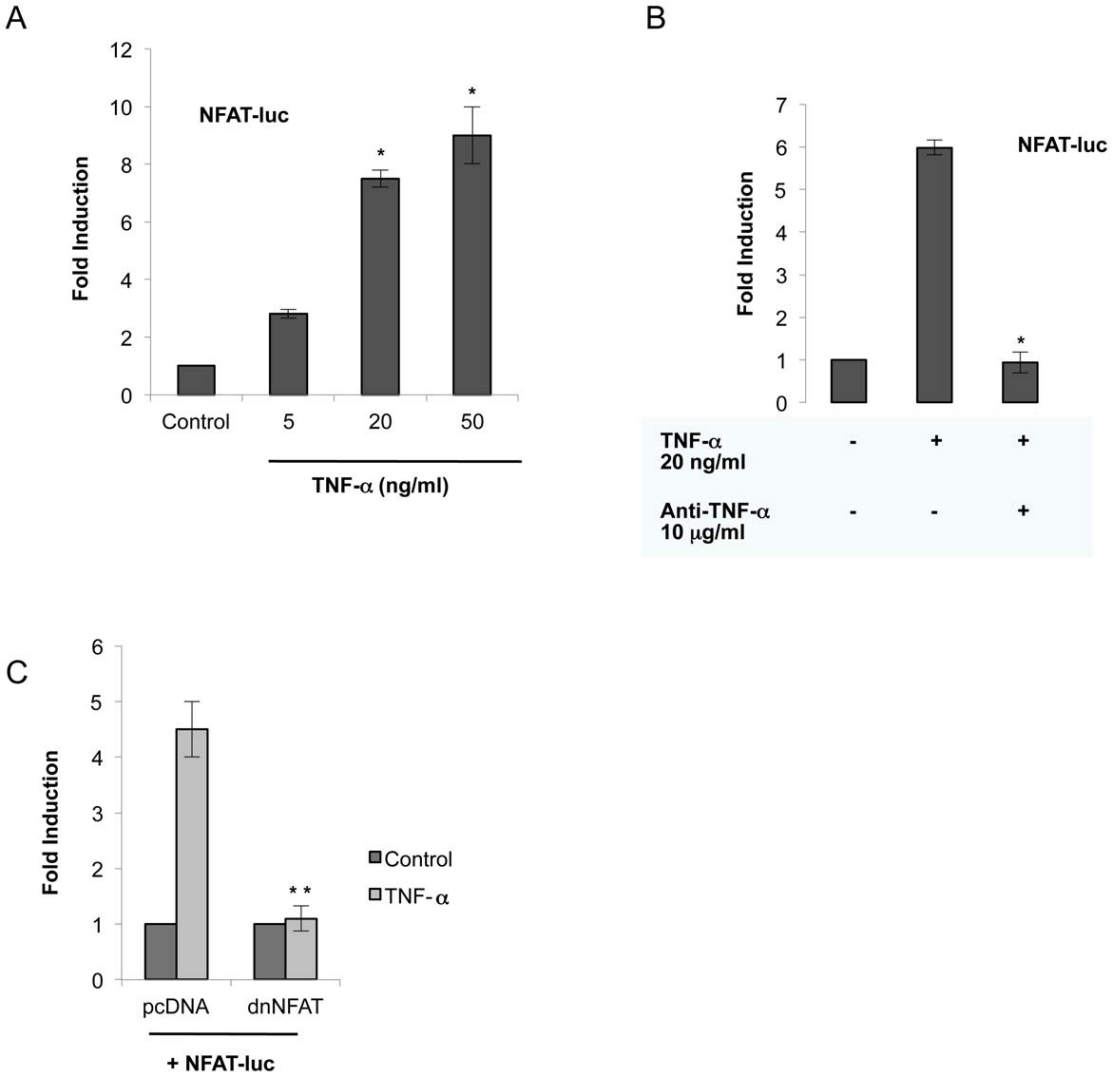
Activation of NFAT activity in human NB cells by TNF-α. A) NB cells were transfected with a NFAT reporter plasmid, and then stimulated by TNF-α at different doses. Luciferase activity was measured 16 h later. Results represent the mean ± SD of three independent experiments performed in triplicate. Significant difference from control: *p<0.01. B) NB cells were transfected with the NFAT reporter plasmid, and treated with TNF-α (20 ng/ml) in the presence of anti-TNF-α (10 µg/ml). Activity of NFAT-luc was measured after 16 h. Data are mean ± SD of three independent experiments performed in triplicate. Significant difference from stimulated cells: *p<0.01. C) SK-N-MC cells were transfected with the NFAT-luc reporter plasmid promoter construct plus an empty vector or an expression vector for a dominant negative (dn) of NFAT. Cells were treated with TNF-α (20 ng/ml) for 16 h and assayed for luciferase activity. Data are from three independent experiments, presented as mean ± SD. Significant difference between stimulated cells: **p = 0.05.

Recently, NFAT activation has been linked in nervous tissue to immune/inflammatory cascades commonly associated with aging and neurodegenerative diseases [17]. To investigate possible changes in the transcriptional activity of NFAT in NB cells treated with TNF-α, we performed transient transfection assays with a reporter plasmid encoding for NFAT. TNF**-**α was able to induce NFAT transcriptional activity in SK-N-MC cells in a concentration-dependent manner (Figure 2A). To ensure the specificity of the effect observed we pretreated NB cultures with anti-TNF**-**α before stimulation with TNF**-**α, and as expected NFAT activity was completely inhibited (Figure 2B). To confirm the role of NFAT on cell death induced by TNF**-**α, we cotransfected the NFAT promoter construct together with a plasmid encoding a version of NFAT (dnNFAT) that lacks the trans activation domain, and acts as dominant negative. Overexpression of this dominant negative version of NFAT completely inhibited the NFAT promoter-driven transcription induced by TNF**-**α in NB cells (Figure 2C).

### TNF-α induces Calcineurin Aα expression but no Ca2+ influx in NB cells

**Figure 3:**
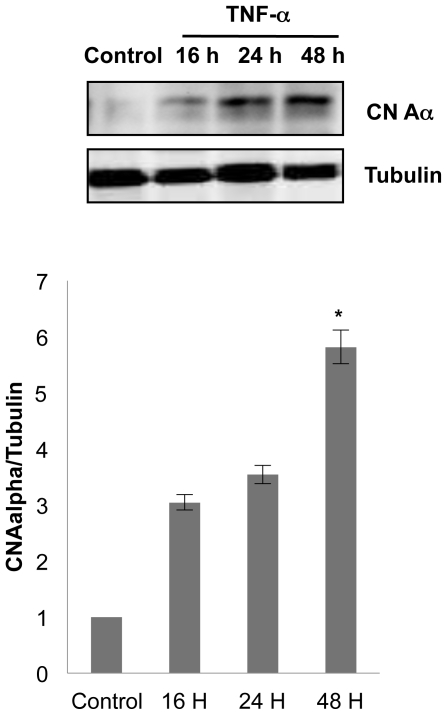
TNF-α induces Calcineurin Aα expression. Top, representative immunoblots probed with antibodies against CaNα in NB cultures at different time set points after TNF-α treatment (20 ng/ml). The membrane was reprobed with α-tubulin antibody to confirm equal protein loading. Bottom, quantitation of CaNα levels was performed by densitometry. Values represent means ± SD of data from two independent experiments. Significant difference from control: *p<0.01.

In an attempt to determine the role of CaN in TNF-α-induced NFAT activity, we investigated its expression in control and stimulated cells. Western blot analysis revealed that TNF-α was able to increase CaNα isoform expression at any time studied being statiscally significant at time of 48 h (Figure 3A).

Probably no signaling molecule is so broad in its distribution yet so specific in its functions as Ca^2+^. Ligand binding of many receptors results in the activation of PLC, release of inositol 1,4,5-trisphosphate (IP_3_), and a transient release of Ca^2+^ from intracellular stores through IP_3_ receptors. However, this initial release of Ca^2+^ is not sufficient to activate NFAT target genes in a number of cell types, and for this an influx of Ca^2+^ through specialized activated Ca^2+^ channels is required. Consistent with previously published data about the presence of different Ca^2+^ channels on SK-N-MC cells [18] was the fact pretreatment of NB cultures with nifedipine (blocker of L-type Ca^2+^ channels) did not alter the actions of TNF-α on NFAT-dependent luciferase activity (Figure S1).

### TNF-α increases NFAT nuclear translocation of NFAT

**Figure 4:**
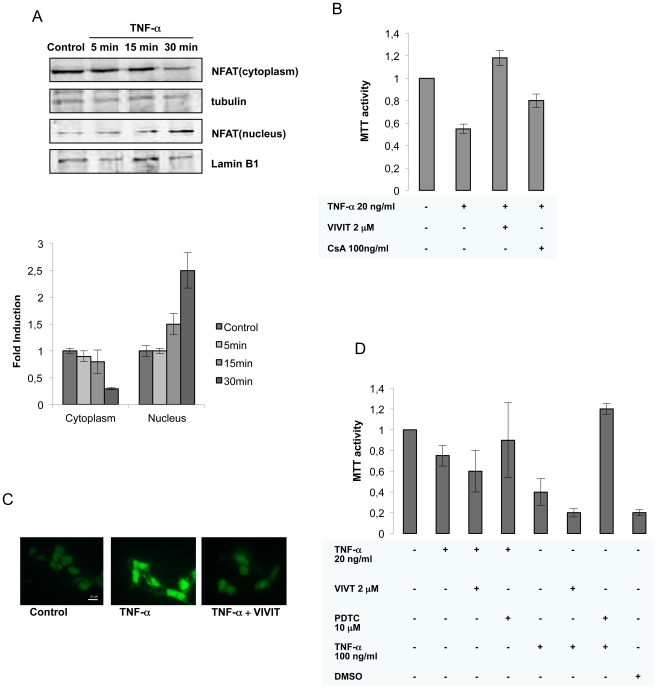
Role of NFAT on TNF-α-mediated cell death. A) Nuclear translocation of NFAT in TNF-α-stimulated SK-N-MC cells. Top, Western blot analysis of fractionated extracts from SK-N-MC cells incubated with TNF-α (20 ng/ml) for the indicated times. NFAT was detected with the anti-NFAT 672 antiserum. Gels shown are representative of 3 independent experiments. Antibodies directed against α-tubulin/Lamin B1 were used as a protein loading control. Each data point is the mean of three replications. Densitometric analysis was used to determine the level of cytoplasmic and nuclear lysates. B) Neuronal death induced by TNF-α is reverted by NFAT inhibition. SK-N-MC cells were untreated (control) or treated with TNF-α (20 ng/ml) alone or in combination with CsA (100 ng/ml) or VIVIT (2 µM) for 24 h before measuring cellular death by MTT. The results represent the means ± SD of three independent experiments performed in triplicate. Significant difference from TNF-α-stimulated cells: *p<0.01. DMSO 10% was used as a positive control of cell death. C) Apoptosis determined by TUNEL. SK-N-MC cells were stained with TUNEL and visualized under a fluorescence microscope with either 100 magnification. Bar: 50 µm. D) Human PBLs were incubated with TNF-α in the presence of VIVIT or PDTC. MTT activity was measured 24 h after. The results represent the means ± SD of three independent experiments performed in triplicate. DMSO 10% was used as a positive control of cell death.

Transcriptional activation by NFAT requires its translocation to the nucleus where it binds to specific recognition sites in the promoter region of target genes. To dissect the mechanism responsible for the TNF**-**α-mediated induction of NFAT activity, we first assessed NFAT dephosphorylation and translocation to the nucleus upon treatment with TNF**-**α. Western blot analysis of subcellular fractions from SK-N-MC cells showed significant differences in the dephosphorylation and translocation to the nucleus of NFAT in stimulated cells 30 min after treatment (Figure 4A). Rapid translocation of NFAT in response to TNF**-**α is consistent with a direct route of activation and suggests that NFAT pathway is an important regulator of TNF**-**α-mediated cascades in SK-N-MC cells.

Stimulation of NFAT does not only involve its nuclear translocation, but also the intrinsic function of the transactivation domain, which is located at the N terminus of NFAT [19]. In this regard, we discarded the regulation of the transactivating function of NFAT by TNF**-**α, with transfection experiments with a Gal4**-**luc reporter plasmid along with a construct (Gal4-NFAT) encoding the N-terminal region of NFAT, where the DNA-binding domain of Gal4 has been joined to the transactivation domain of NFAT (Figure S2). These results suggest that regulation of NFAT by TNF-α in human NB cells depends primarily on the NFAT promoter region.

Upon activation, nuclear NFAT is able to increase transcription of genes through still not well-understood mechanisms mediated by its transactivation domain in such a way that stimuli leading NFAT translocation and DNA binding were also able to induce transactivation mediated by the N-terminal transactivation domain (TAD) of NFAT proteins [20], [21]. Thus, we tested whether TNF-α had an effect on this step in the NFAT signaling pathway. For this, SK-N-MC cells were co-transfected with a construct fusing the transactivation domain of the NFATc2 (1–415) to the Gal4 DNA binding domain (DBD) along with a Gal4-luc reporter. When the ability of TNF-α stimulation to increase the transactivation of Gal4-NFAT TAD was measured, we did not find any change in transactivation activity on stimulated cells (Figure S2).

Since our results suggest that cell death in SK-N-MC cells requires NFAT transcription factor which is regulated by the phosphatase CaN, we therefore analyzed the levels of neurotoxicity in TNF-α stimulated SK-N-MC cells in the presence of the CN inhibitor cyclosporin A (CsA) (100 ng/ml) or VIVIT (2 µM), a peptide that prevents CN from docking to and dephosphorylating NFAT transcription factors. Pretreatment with either CsA or VIVIT for 1 h before TNF-α stimulation led to a decrease of neuronal toxicity, consistent with the notion that this process requires NFAT (Figure 4B). These data were confirmed by TUNEL (Figure 4C). In contrast, VIVIT had no effect on viability of TNF-α-stimulated lymphocytes, a process that has been shown to be NF-κB dependent (Figure 4D).

Although it has been previously described p53-mediated dependent and independent pathways in TNF**-**α**-**induced apoptosis of human brain cells [22], here we did not detect any variation in p53-Ser46 expression after TNF**-**α treatment in human NB cells (Figure S3).

### Functional significance of FasL expression by NB cells

**Figure 5:**
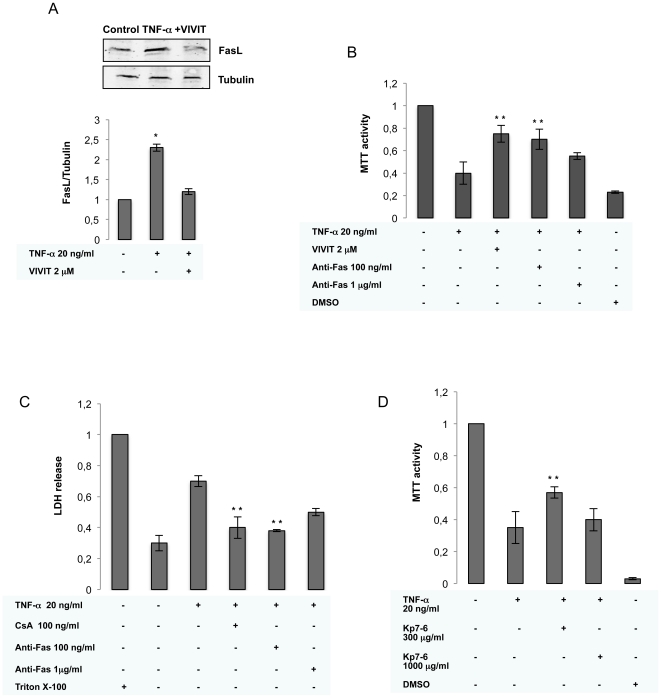
FasL induction by TNF-α in SK-N-MC cells. **A**) The level of FasL is increased in TNF-α treated NB cells. Protein lysates were prepared from NB cells 16 h after TNF-α stimulation. Top, lysates were analyzed by Western blot analysis by using antibody directed against FasL. Antibody directed against α-tubulin was used as a protein loading control. Bottom, densitometric analysis was used to determine the level of FasL expression in NB cells, with values normalized to tubulin levels. Error bars represent standard errors of the means. Significant difference from untreated cells: *p<0.01. B) SK-N-MC cells were pretreated with antiFas at the indicated doses before stimulation with TNF-α (20 ng/ml). Cell viability was determined using the MTT and LDH assays (C). The results represent the means ± SD of three independent experiments performed in triplicate. Significant difference from stimulated cells: **p = 0.01. D) Inhibition of TNF-α-induced apoptosis in SK-N-MC cells by the antagonistic peptide Kp7-6. SK-N-MC cells were stimulated with TNF-α in the presence or absence of Kp7-6 at the indicated doses. MTT activity was measured 24 h later. **p = 0.02.

CaN-dependent activation of NFAT has been shown to result in upregulation of the death receptor ligand FasL [23]. FasL binds to its receptor Fas and triggers the Fas/FasL apoptotic death cascade that results in cleavage of DNA. To investigate whether TNF-α-induced activation of NFAT and subsequent apoptosis of SK-N-MC cells is linked to the Fas/FasL death pathway, we examined the expression of FasL after TNF**-**α stimulation. TNF-α upregulated FasL expression after 16 h of stimulation, and more importantly, VIVIT inhibited TNF-α-induced FasL protein by 50% (Figure 5A), suggesting that NFAT activation is required for TNF-α-mediated FasL expression in NB cells. More important, pretreatment with Abs specific for Fas significantly blocked the potentiation of cell death by TNF**-**α stimulation measured by MTT (Figure 5B), and LDH release (Figure 5C). Likewise, the antagonist Kp7-6, which binds FasL and inhibit Fas/FasL interaction, diminished toxicity induced by TNF**-**α in NB cells (Figure 5D).

### Ingenuity Pathways Analysis of the newly identified TNF-α regulated genes

**Figure 6:**
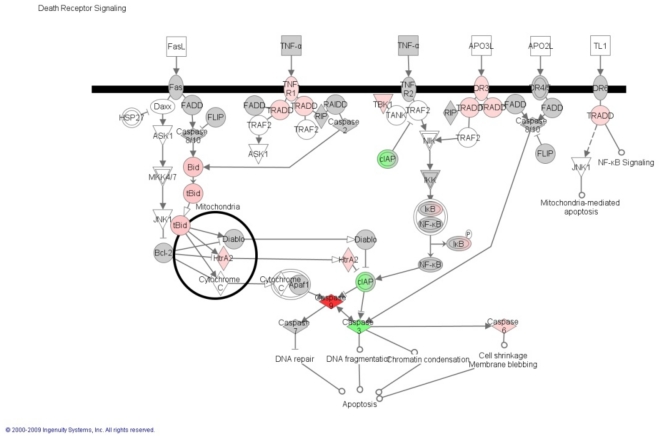
The pathway was assembled using Ingenuity Pathways Analysis (Ingenuity Systems;www.ingenuity.com **), and it shows genes involved in both the extrinsic and intrinsic mechanisms of apoptosis.** Official gene symbols are used. Upregulation is represented by red, and downregulation is represented by green. Color intensity corresponds to the degree of differential regulation.

Having demonstrated that TNF**-**α induces caspase-3-independent cell death in NB cells, we assessed more globally the mechanisms involved in this TNF-α-induced cell death using microarray analysis. Therefore, RNA was prepared from unexposed SK-N-MC cells and exposed to TNF**-**α for 16 and 24 h. Three biological replicates were performed per group. To evaluate further the functional pathways in which the newly identified TNF**-**α-regulated genes are involved in SK-N-MC cells we used the Ingenuity Pathway Analysis (IPA) system. BIRC7 and TNFRSF1B were upregulated after 16 h. At this time, BIRC3, RELB, and NFKB1 were the only downregulated genes detected in this analysis (Table 1). More interesting was the analysis performed after 24 h where differential gene expression analysis revealed 22 modulated transcripts (6 decreased and 16 increased) in TNF-α-stimulated cells compared with controls. A partial list of the most relevant genes is shown in Table 2. Data were normalized using the glyceraldehyde-3-phosphate dehydrogenase (GAPDH) gene as internal control. The cDNA array approach was used to identify genes of which transcripts might be regulated in NB cells after stimulation with TNF-α. We used the pathway drawing function of Ingenuity Pathways Analysis to display gene expression changes related to apoptosis, as is shown in Figure 6. Intriguingly, the majority of the upregulated genes were associated with the intrinsic mechanism of cell death (HIP1, PYCARD, caspase-9). Conversely, several components of the extrinsic pathway were uniquely upregulated (member 1B of the TNFR family (TNFRSF1B), member 25 of the TNFR family (TNFRSF25), and member 1A of the TNFR family (TNFRSF1A)).

**Table 1:** Differentially expressed genes in SK-N-MC cells 16 h post-treatment with TNF-α.

Gene Symbol	Description	Log ratio up-regulated
**BIRC7**	baculoviral IAP repeat-containing 7	3,5
**TNFRSF1B**	tumor necrosis factor receptor superfamily, member 1B	2,4
		**Log Ratio down-regulated**
**BIRC3**	baculoviral IAP repeat-containing 3	−2,7
**RELB**	v-rel reticuloendotheliosis viral oncogene homolog B	−2,2
**NFKB1**	nuclear factor of kappa light polypeptide gene enhancer in B-cells 1	−2,1

**Table 2:** Differentially expressed genes in SK-N-MC cells 24 h post-treatment with TNF-α.

Gene Symbol	Description	Log ratio up-regulated
**CASP9**	Caspase-9, apoptosis-related cysteine peptidase	11,4
**PYCARD**	PYD and CARD domain containing	6,2
**CASP1**	Caspase-1, apoptosis-related cysteine peptidase	4,7
**HIP1**	huntingtin interacting protein 1	3,4
**DAPK1**	death-associated protein kinase 1	3,1
**BID**	BH3 interacting domain death agonist	3,1
**BCL3**	B-cell CLL/lymphoma 3	3,0
**BIRC7**	baculoviral IAP repeat-containing 7	2,8
**BOK**	BCL2-related ovarian killer	2,8
**CASP6**	Caspase-6, apoptosis-related cysteine peptidase	2,5
**HTRA2**	5-hydroxytryptamine (serotonin) receptor 2A	2,4
**BAD**	BCL2-associated agonist of cell death	2,3
**TBK1**	TANK-binding kinase 1	2,3
**TRADD**	TNFRSF1A-associated via death domain	2,2
**TNFRSF25**	tumor necrosis factor receptor superfamily, member 25	2,2
**TNFRSF1A**	tumor necrosis factor receptor superfamily, member 1A	2,0
		**Log Ratio down-regulated**
**BCL2L11**	BCL2-like 11 (apoptosis facilitator)	−4,7
**BIK**	BCL2-interacting killer (apoptosis-inducing)	−3,9
**CASP3**	Caspase-3, apoptosis-related cysteine peptidase	−2,7
**BCL10**	B-cell CLL/lymphoma 10	−2,5
**BIRC2**	baculoviral IAP repeat-containing 2	−2,3
**CARD9**	caspase recruitment domain family, member 9	−2,1

### TNF-α causes the release of cytochrome c from the mitochondria, and induces cleavage of 35-kDa active caspase-9 in NB cells

To determine which caspases are activated in TNF-α-induced cell death, we stimulated NB cultures for different times, and cell lysates were prepared to assess caspases activities.

**Figure 7:**
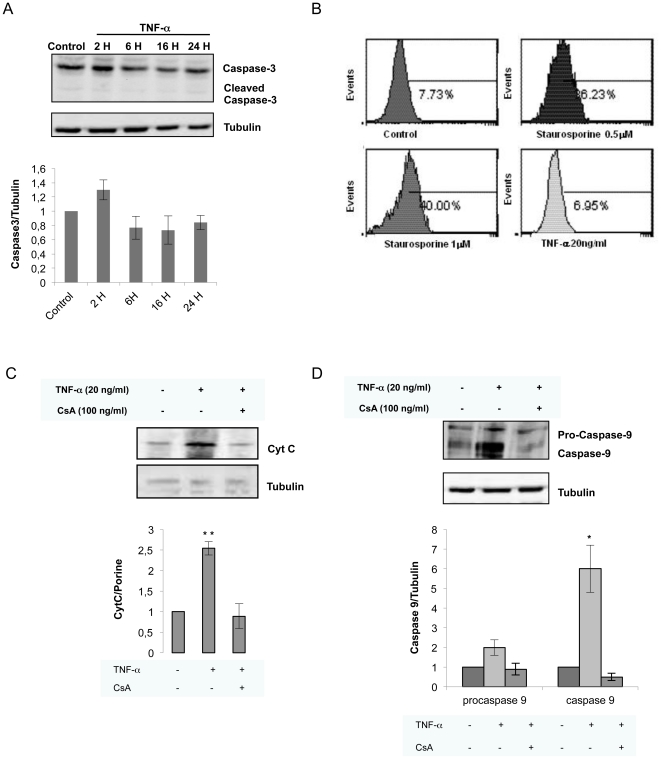
Effect of TNF-α on cytochrome c release, and cleavage of caspase-9 in SK-N-MC cells. **A**) NB cells were incubated or not with TNF-α for the indicated times, and total extracts were immunoblotted with caspase-3 Ab. B) Caspase-3 was measured after 24 h of treatment by flow cytometry. Treatment with staurosporine 0.5-1 µM for 3 h was used as positive control. C) NB cells were incubated with TNF-α in the presence of CsA for 24 h. The mitochondria free cytosolic fractions were prepared for Western blot analysis as described under Experimental Procedures. α-tubulin was used as cytosolic marker and as control for protein loading. The graphs show the mean increase in cytochrome c/caspase-9 (D) activity levels in TNF-α treated cells compared to that of unstimulated controls. Porine antibody was used as a control to ensure proper fractionation and loading of mitochondrial pellet. Error bars represent standard errors of the means. Significant difference from stimulated cells: *p<0.01; **p = 0.01.

Caspase-3 appears to be an essential component of the apoptotic machinery in many cell types and a key player in many types of neuronal apoptosis [24]. We analyzed the ability of TNF-α to trigger the activation of caspase-3 activity in TNF-α-stimulated cells versus control ones. In contrast with expected results, we did not observe significant activation of caspase-3 in SK-N-MC cells after TNF-α treatment (Figure 7A, B).

Damage to the mitochondria in other paradigms has been demonstrated to result in the release of mitochondrial cytochrome c into the cytoplasm and subsequent activation of caspase-9, which in turn can elicit the activation of caspase-3 [25]. To evaluate whether mitochondrial release of cytochrome c is involved in TNF-α-induced cell death, we prepared cytosolic and mitochondrial extracts from NB cells at various time points after treatment, and cytochrome c protein levels were measured by immunoblot analysis. Cytosol from untreated cells did not contain any detectable cytochrome c protein (data not shown). In contrast, cytosolic cytochrome c accumulated significantly in TNF-α-stimulated NB cells (Figure 7C). Along with mitochondrial cytochrome c release, caspase-9 activity was increased by approximately 2-fold at 24 h after the induction of apoptosis by TNF-α stimulation; this increase in activity was found to be inhibited greatly in the presence of VIVIT suggesting that NFAT signaling is, at least in part, responsible for its activation (Figure 7D).

## Discussion

Inflammation is a cardinal host response to injury, tissue ischemia, autoimmune responses or infectious agents. Although most inflammatory mediators have relatively few actions in healthy CNS tissue being expressed at very low, or undetectable, levels, they are induced rapidly in response to tissue injury or infection, and exert diverse actions [1].

TNF-α is released during various inflammatory diseases of the CNS, being synthesized by microglia, astrocytes, and some populations of neurons [26], [27]. Moreover, since TNF-α receptors are widely expressed by neurons, astrocytes and microglial cells both direct and indirect actions of TNF-α on neurons must be considered. The experiments in this study, although performed in cell cultures, reflect physiological conditions within the brain during inflammatory CNS diseases. So, when we investigated its effect on NB cultures, we observed an increase in cell death in absence of glia cells, and more important was the fact that TNF-α was able to induce an increase of NFAT transcriptional activity in NB cells. The specific nature of NFAT activity after TNF-α treatment was further supported by the fact that pretreatment with anti-TNF-α as well as dnNFAT overexpression were enough to completely block the NFAT activation in treated cells. In addition to NFAT phosphorylation, entry into the nucleus and binding to DNA, NFAT-dependent transcriptional up-regulation involves activation of the intrinsic transactivation activity mediated by the N-terminal TAD of NFAT [20], [21]. Here we have observed that transcriptional activity induced by the Gal4-NFAT fusion protein was not affected by treatment with TNF-α. The inability of TNF-α to stimulate the Gal4-NFAT strongly suggests that the transactivation of NFAT is unlikely to be responsible for the effects observed in this paper indicating that the regulation of NFAT by TNF-α depends primarily on the NFAT promoter region.

NFAT proteins are specialized to sense and respond to dynamic changes in intracellular calcium concentration because of their ability to rapidly translocate to and from the nucleus via the opposing activities of CaN and kinases [28]. Calcium/calmodulin activation of the CaN results in dephosphorylation of target proteins [29]. Dephosphorylation of NFATc leads to exposure of a nuclear localization sequence, translocation from the cytoplasm to the nucleus [30], and transcription of specific target genes. CaN is inhibited by drugs such as CsA and FK506 through the binding of these drugs to their appropriate receptors (immunophilins) [31]. Although initially used after organ transplantation because of their immunosuppressive properties, immunophilin ligands have been a center of attention as a putative therapeutic strategy for neuroregeneration and neuroprotection [32], [33]. In this regard, it has been previously described a direct role of calcineurin in neuroprotection in animal models [34].

Recently Hui *et al* have described, that direct inhibition of CaN is neuroprotective in vivo [35], and Sama *et al* have demonstrated that specific NFAT inhibition provides nearly complete protection against IL-1β dependent toxicity [36]. Consistent with this, we found that TNF-α increased CaN expression in treated NB cells. Although the aim of the study was not to determine the mechanism of CaN induction by TNF-α, we can suppose that given the protracted of induction the response is quite indirect. Interestingly, the TNF-α-induced apoptosis was shown to be partly dependent on activation of NFAT as suggested by the significant attenuation of apoptosis with VIVIT treatment.

It has been previously described that expression of the membrane-bound death receptor ligand FasL is mediated by NFAT [37]. When FasL binds to its receptor Fas, the intracellular machinery associated with the death receptor Fas is activated and eventually leads to apoptosis by caspase activation and subsequent DNA cleavage [23]. Moreover, FasL and Fas receptors are widely expressed in the nervous system in both neurons and glial cells [38], [39]. Here, we show that TNF-α treatment also increased FasL levels in NB cells, and this increase was suppressed by VIVIT. Moreover, cell death was completely abrogated in the presence of different Fas/FasL antagonists. So it is possible that upregulation of FasL results in TNF-α-induced death of NB cells by inserting into the cell membrane and binding to its receptor Fas, which is expressed on the membranes of adjacent neurons, and subsequently triggering apoptosis.

Classically, the role of NFAT is described in the context of lymphocyte function and its role in the immune system. Alternatively, this study provides an example of a role for NFAT in the context of the nervous system, a role that is becoming more common. Collectively, our results indicate that NFAT is activated after TNF**-**α treatment and that this event leads to increased FasL expression and apoptosis of NB cells.

TNF**-**α family ligands are the main inducers of apoptosis in the CNS and thus contribute to brain injuries in many neurological diseases. Although many stimuli exist, the final phases of apoptosis are executed by a few common effector caspases. In nonneuronal cells, mitochondria have been shown to accelerate activation of caspases by releasing proapoptotic molecules, cytochrome c, and the apoptosis-inducing factor. The extrinsic pathway of apoptosis can be induced through oligomerization of death receptors such as Fas, TNFR, DR3, TRAIL-R4, and TRAIL-R5 after engagement with their respective ligands. This oligomerization, in turn, results in recruitment of adaptor proteins and activation of caspase cascades. Initial activation of caspase-8 stimulates apoptosis in two ways: it can directly cleave and activate caspase-3 or, alternatively, it can cleave Bid, a proapoptotic Bcl2 family member. This cleaved (or truncated) bid (tBid) is translocated to mitochondria, inducing cytochome c release, sequentially activating caspases-9 and -3, and resulting in DNA fragmentation and cell death [40].

Caspase-8 is another caspase that, like caspase-9, appears to be activated upstream of caspase-3 and in addition has been implicated possibly to play a role in the cell death associated with neurodegenerative paradigms. Although in array analysis we did not detect caspase-8 activation, we found an increase of expression of Bid protein, a Bcl-2 family member, which has been shown to be a specific substrate of caspase-8 and to play a role in caspase-8-mediated mitochondrial damage and cell death [41]. It is possible that caspase-8 activation occurs early in the process of cell death and that 16 h is relatively late time point for early gene expression. Furthermore, TNF**-**α significantly induced the cytosolic release of cytochrome c which leads to activation of caspase-9, providing evidence that cell death is mitochondria dependent.

It is important to note that we have found increased several proapoptotic proteins as HIP-1, which has been shown to activate caspase-9, DAPK1 which is/calmodulin-dependent serine/threonine kinase, or Pycard which was upregulated 6-fold in TNF**-**α**-**stimulated cells, and has been shown to associate with Bax, a key protein in the apoptotic cascade that induces cytochrome c release from the mitochondria [42]. This effect ultimately leads to activation of caspase-9, which also is upregulated more than 11-fold.

Surprisingly, the treatment with TNF-α had no effect on caspase-3 activation. Previously Kuida et al demonstrated a caspase-9 dependent and caspase-3 independent apoptotic pathway, since thymocytes from caspase-9^-/-^ mice are resistant to gamma irradiation and dexamethasone, whereas thymocytes from caspase-3^-/-^ mice are sensitive, suggesting that other caspases are directly activated by caspase-9 [43]. Future studies are necessary to elucidate the downstream target of caspase-9.

Moreover, we detected upregulation of proteins as BOK, and BAD. BOK promotes both caspase-dependent and caspase-independent apoptosis at the level of mitochondria in various cell types by promoting the release of proapoptotic mitochondrial factors to the cell cytosol. Once BAD is dephosphorylated (posttranslational modification), it is active; it translocates to the outer membrane of the mitochondria, and forms heterodimers with BCL-XL to block BCL-XL antiapoptotic function.

**Figure 8:**
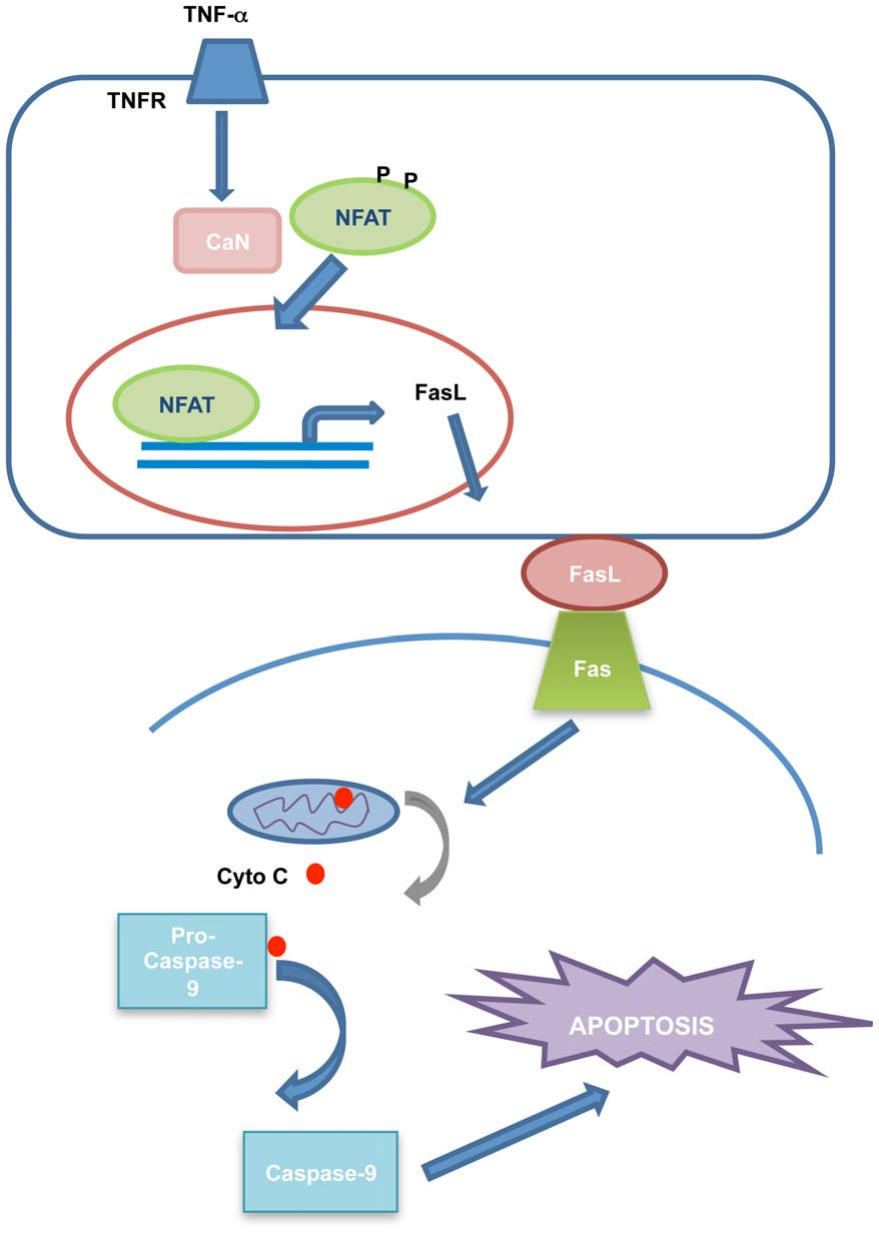
A proposed model for TNF-α-induced cell death in NB cells.

Due to the huge impact of TNFR modulation on many disorders, the potential benefits of specific, targeted TNFR therapeutics even for CNS diseases seem likely. Therefore, targeting components of downstream signaling scaffolds or specific TNF receptor conformations associated with a particular downstream pathway provides the opportunity to block the pathogenic component of TNF**-**α signaling while preserving the beneficial component. Nevertheless, the neuroprotective effects of NFAT inhibition observed here suggest that NFAT may be a promising molecular target for the treatment of several neurodegenerative diseases. The proposed TNF-α-induced cell death signaling pathway mediated by NFAT is shown in Figure 8.

## Materials and Methods

### Cell culture and treatments

The NB cell line, SK-N-MC (from ATCC HTB10), was routinely grown in RPMI 1640 (Biochrom KG Seromed, Berlin, Germany) containing 10% heat-inactivated fetal calf serum, 1% penicillin/streptomycin, and 2 mM L-glutamine at 37°C and 5% CO_2_.

RhTNF-α was from Promega (Promega Corporation, WI, USA). Anti-human TNF-α neutralizing antibody was purchased from R&D Systems (Europe, Abingdon, UK). CsA was purchased from Sigma (St. Louis, MO). Pyrrolidine dithiocarbamate (PDTC) (an NF-κB inhibitor), and nifedipine were from Santa Cruz Biotechnology (Inc. CA, USA). Antibodies specific for Fas were from ENZO (Alexis). 11R-VIVIT (NFAT inhibitor cell permeable), and the Fas/FasL antagonist, Kp7-6, were from Calbiochem, (Merck KGaA, Darmstadt, Germany). Purified mouse anti-cytochrome c monoclonal antibody was from BD Pharmingen (BD Biosciences Pharmingen, Becton Dickinson France).

### Plasmid Constructs

The pNFAT-luc reporter plasmid was a gift from Dr. G. R. Crabtree (Department of Pathology, Howard Hughes Medical Institute, Stanford University Medical School, Stanford, CA 94305, USA). It contains three tandem copies of the distal NFAT site of the human IL-2 promoter fused to the minimal human IL-2 promoter. The dominant negative NFATc1 (pSH102C.....418), was generously provided by Dr. G.R. Crabtree. The Gal4-hNFATc2(1–415) construct contains the first 415 amino acids of the human NFATc2 fused to the DNA binding domain (DBD) of the yeast Gal4 transcription factor (amino acids 1–147) [19] in the parental Gal4-DBD plasmid. The Gal4-luc reporter plasmid includes five copies of Gal4 DNA binding sites fused to the luciferase gene.

### Transcription Assays

Transcriptional activity was measured using luciferase reporter gene assays in transiently transfected cells by Lipofectin reagent as recommended by the manufacturer (Life Technologies, Inc., Grand 8 Island, NY, USA). In cotransfection experiments, 0.15-0.5 µg/ml of the correspondent expression plasmid was included. The total amount of DNA in each transfection was kept constant by using the corresponding empty expression vectors. Protein contents were measured using the bicinchoninic acid method (BCA protein assay kit from Pierce, Rockford, IL, USA) according to the manufacturer's instructions. Luciferase activity was determined by using a luciferase assay kit (Promega, Madison, WI, USA) with a luminometer 1450 Microbeta Luminiscence Counter. The efficiency of transfection was measured using the pSV-β-Galactosidase Control Vector Kit (Promega Corporation Madison) following manufactures indications. The data presented are expressed as fold induction respect untreated cells of at least 3 independent experiments.

For the transactivation assays, cells were cotransfected with 10 ng of the Gal4DBD-NFAT construct and 100 ng of 5XGal4 luciferase reporter and cultured with or without TNF-α (20 ng/ml). Reporter activity is expressed as fold induction above control.

### Western blot analyses

Cells were exposed to different stimuli, washed with phosphate-buffered saline (PBS) and lysed with buffer lysis. Protein contents were measured using BCA protein assay according to the manufacturer's instructions. Samples were separated into a 10-15% SDS polyacrylamide gel and blotted onto a polyvinylidene fluoride (PVDF) membrane (Millipore, Bedford, MA, U.S.A.) by semidry transference blotting. Membranes were blocked overnight at 4°C using Rotiblock (Roth, Karlsruhe) before incubation with the primary antibody. Rabbit anti-Calcineurin A polyclonal antibody (Stressgen, MI, USA), phosphor-p53 (Ser46), FasL antibody, Caspase-9 (C9) mouse mAb, and Caspase-3 (3G2) mouse Ab (Cell Signaling Technology, Inc), anti-porine (a gift of Dr. Mar González, CIB-CSIC, Madrid, Spain) were used as primary antibodies as appropriate. To detect NFAT nuclear translocation, nuclear and cytosolic protein extracts were obtained using Nuclear/Cytosolic fractionation kit (MBL International). After SDS-PAGE and blotting as above, cells were incubated with an anti-NFATc2 serum (gift from Dr. J. M. Redondo, Centro de Biología Molecular, Universidad Autónoma, Madrid, Spain) (1/3000) for 2 h at room temperature. Membranes were washed and incubated with horseradish peroxidase-conjugated secondary antibody (Amersham-Pharmacia Biotech; 1∶10000) for one hour at room temperature. Proteins were detected using the enhanced chemiluminescence system (Amersham-Pharmacia Biotech).

In all cases, equal amounts of total protein were analyzed across groups. We used α-tubulin (Sigma, St. Louis, MO), and Lamin B1 (Santa Cruz Biotechnology Inc), as loading control for total/cytoplasmatic, and nuclear extracts respectively.

### Cytochrome c Release Assays

Cells were harvested, resuspended in 1 ml of lysis buffer (10 mM Tris, 250 mM Sacarosa, 1 mM EDTA, and complete proteinase inhibitors (Roche Applied Science)), and lysed by freeze/thaw, and centrifuged at 750× g for 10 min. The supernatant was centrifugated again at 10000×g for 20 min. Supernantants, containing cytosolic proteins were analyzed by Western blot.

### RNA preparation

RNA from SK-N-MC cells cultured with or without TNF-α (16 or 24 h), was prepared using RNAEasy mini kits (Qiagen).

### Measurement of cell death and apoptosis

Neuronal cell viability was assessed by the release of lactate dehydrogenase (LDH) into the culture medium, which indicates loss of membrane integrity and cell death. LDH activity was measured using a commercial kit (Cytotoxicity Detection Kit (Roche Applied Science)) according to manufacturer's protocol. Percent cell death is determined by the amount of LDH measured in the medium divided by the amount of LDH after addition of 1% Triton-X 100.

### MTT assay

Mitochondrial activity (a measure of cellular viability) was measured with the MTT assay (Sigma, St. Louis, MO) as per manufacturer's protocol.

### Fragment end labeling of DNA (TUNEL)

Fragmented DNA was detected *in situ* by the terminal deoxynucleotidyl transferase (TdT)-mediated binding of 3′-OH ends of DNA fragments generated in response to apoptotic signals, using a commercially available kit from Roche as per manufacturer's protocol.

### Taqman Human Apoptosis Array-Real-Time RT-PCR

RNA (1 µg) from NB cells exposed or not to TNF-α was used to generate cDNA and then assessed by TaqMan assays (Applied Biosystems, Darmstadt, Germany). Briefly, random hexamers were used to prime RNA samples for reverse transcription using MultiScribe (Applied Biosystems, Darmstadt, Germany) reverse transcriptase, after which PCR products for all the genes tested in this report were assessed in triplicate wells using TaqMan predeveloped assay reagents. The assay contains for 93 human genes in addition to 3 endogenous controls (18S, ACTB, GAPDH). Relative transcript levels were determined by the following formula: 1/(ΔCt target-ΔCt control), where Ct is the threshold cycle during the exponential phase of amplification. Real-time quantitative RT-PCR was performed on an ABI 7900HT system (Applied Biosystems, Darmstadt, Germany).

### Microarray data processing

Real-time PCR data were analyzed using the Sequence detector version 2.2.2 software supplied with the 7900 HT Fast-Real-Time PCR System (Applied Biosystems, Darmstadt, Germany). Relative expression of the transcripts was measured in the TaqMan 7900 HT Fast Real-Time PCR system (Applied Biosystems, Darmstadt, Germany). Stimulated samples were normalized to the corresponding medium-only control. Normalized data were then imported and analyzed using Ingenuity Pathway Analysis software (Ingenuity Systems, Mountain View, CA, USA). Pathway analysis revealed biologic pathways altered in SK-N-MC cells following exposure to TNF-α. Next, the raw expression values for experimental (TNF-α exposed) and control (medium only) were compared to identify those genes with the greatest difference. Those genes detected were analyzed further by Western blot.

### IPA: Network, gene ontology, and canonical pathway analysis

Unsupervised cluster analysis gene accession numbers were imported into the version 3.1 IPA software (Ingenuity Systems, Mountain View, CA, USA) and the gene products were categorized based on location, cellular components, and reported or suggested biochemical, biologic, and molecular functions using the software. The Ingenuity Pathways Knowledge Base is currently the world's largest database of knowledge on biological networks. We exploited this database to define the presence of functional associations within the genes detected by microarray analysis and to identify differences between the ontological gene classes that were enriched among differ expressed genes. This ontological gene classification provides the controlled vocabulary to describe gene and gene product attributes.

### Statistical analysis

The data were expressed as the mean ± SD from three to six independent experiments. Statistical significance between groups was determined by two-tailed Student's t test. Differences were considered significant when p<0.05.

## Supporting Information

Figure S1 **L-VSCCs are not involved in CaN/NFAT activation in NB cells in response to TNF-α.** NB cells were transfected with NFAT reporter plasmid and pretreated with nifedipine (10 µM) at the indicated times before TNF-α stimulation. Luciferase activity was measured 16 h later. Results are the mean ± SD of four different experiments and fold induction was normalized to TNF-α treated cells.
(TIF)

Figure S2 **TNF-α does not induce any increase of NFAT transactivation.** Cells were cotransfected with 10 ng of the Gal4DBD-NFAT construct and 100 ng of 5XGal4 luciferase reporter and cultured with medium in basal condition or cultured with TNF-α (20 ng/ml). Reporter activity is expressed as fold induction above control. Values represent means ± SD of triplicate cultures.
(TIF)

Figure S3 **P53 is not involved in TNF-α-mediated cell death**. p53-PSer46 protein levels were determined by Western blot analysis. Cell lysates from untreated or stimulated cells for the indicated times were separated by SDS-PAGE on 10% acrylamide gel, blotted, and incubated with antibodies against p53-PSer46.
(TIF)
