# Supplementary figures and images for: TNF-α Contributes to Caspase-3 Independent Apoptosis in Neuroblastoma Cells: Role of NFAT
Source: PLoS One. 2011 Jan 27;6(1):e16100. doi: 10.1371/journal.pone.0016100 (PMC3029262; doi:10.1371/journal.pone.0016100)

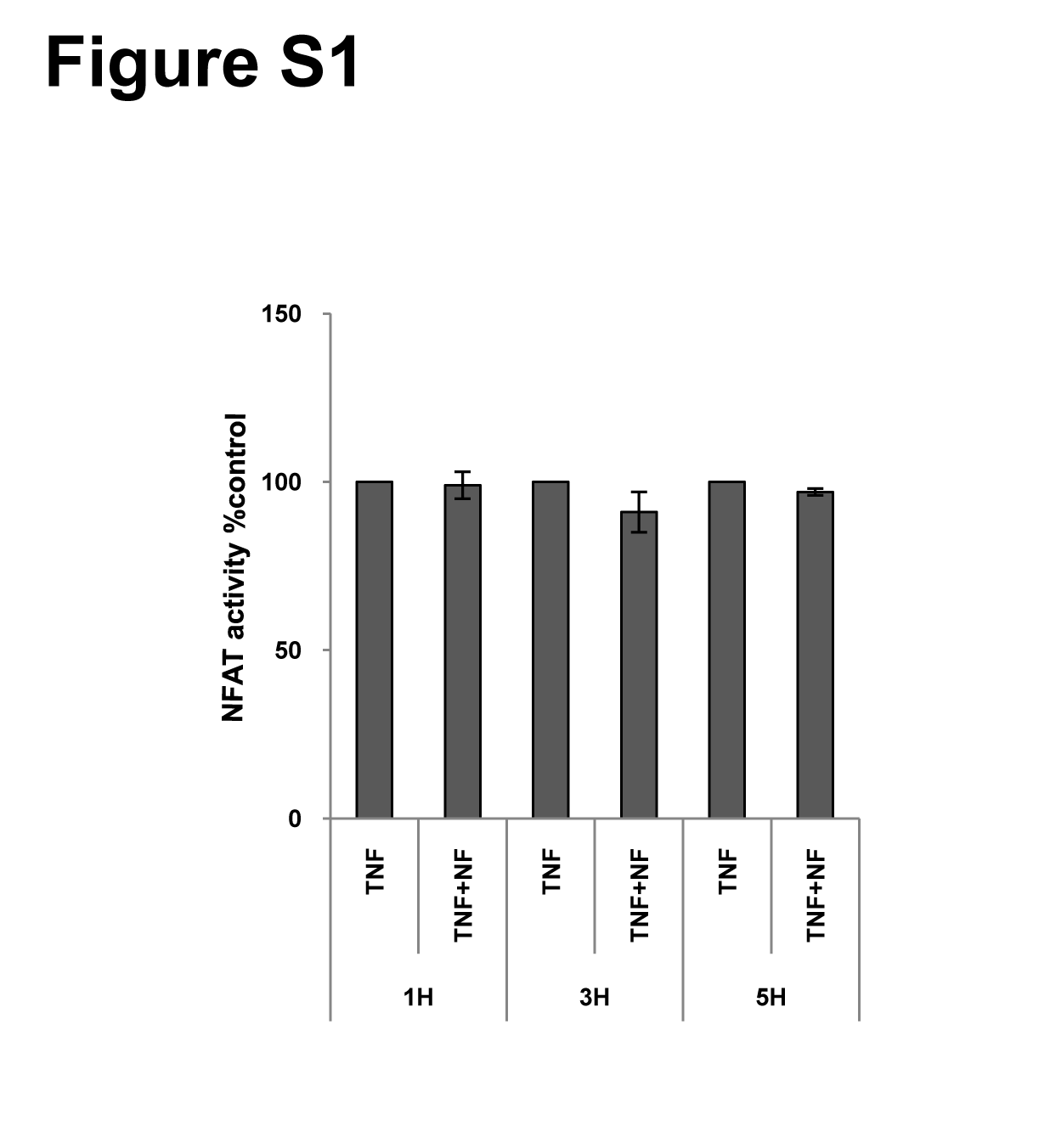

Supplement: Figure S1 — L-VSCCs are not involved in CaN/NFAT activation in NB cells in response to TNF-α. NB cells were transfected with NFAT reporter plasmid and pretreated with nifedipine (10 µM) at the indicated times before TNF-α stimulation. Luciferase activity was measured 16 h later. Results are the mean ± SD of four different experiments and fold induction was normalized to TNF-α treated cells. (TIF) [file pone.0016100.s001.tif]

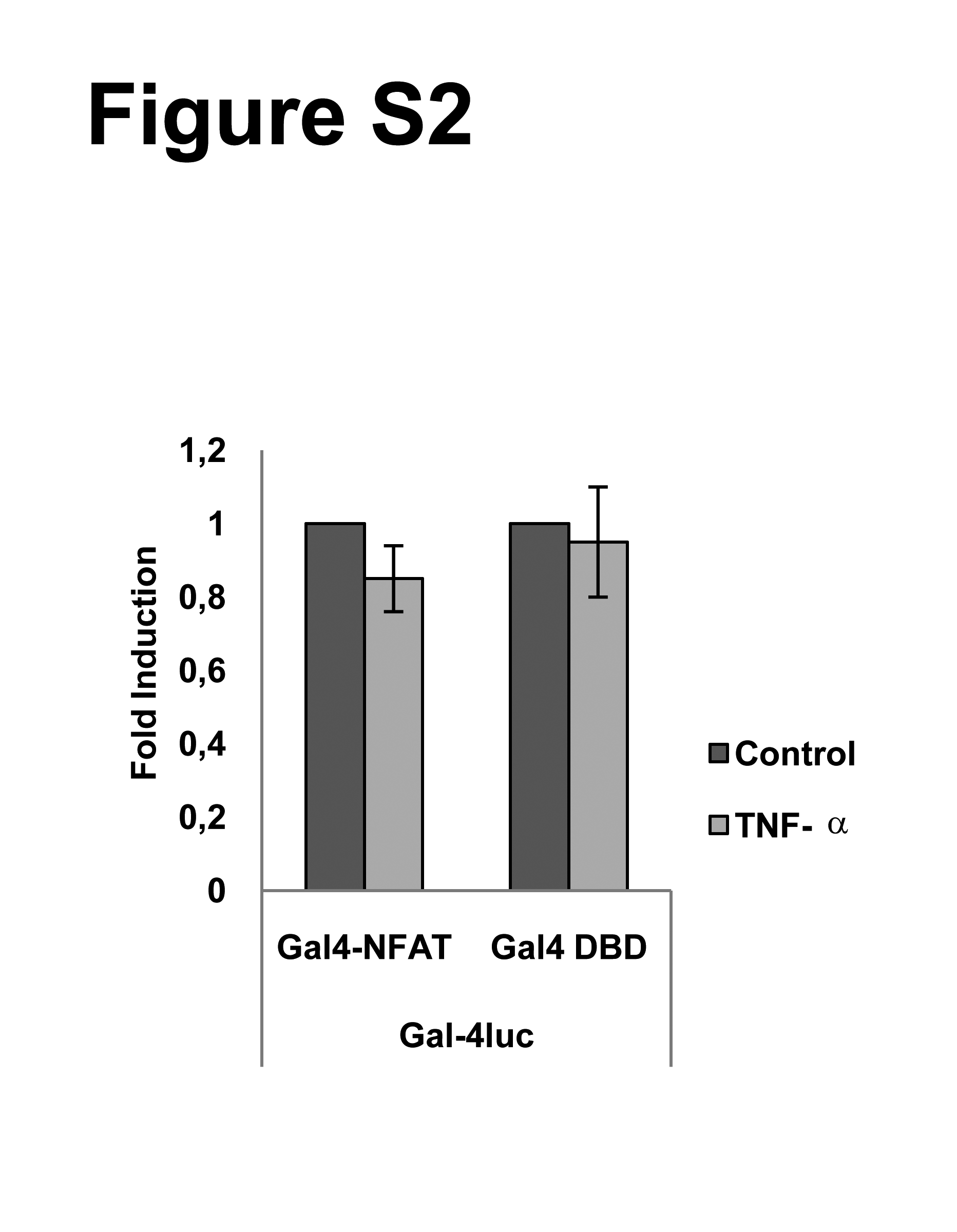

Supplement: Figure S2 — TNF-α does not induce any increase of NFAT transactivation. Cells were cotransfected with 10 ng of the Gal4DBD-NFAT construct and 100 ng of 5XGal4 luciferase reporter and cultured with medium in basal condition or cultured with TNF-α (20 ng/ml). Reporter activity is expressed as fold induction above control. Values represent means ± SD of triplicate cultures. (TIF) [file pone.0016100.s002.tif]

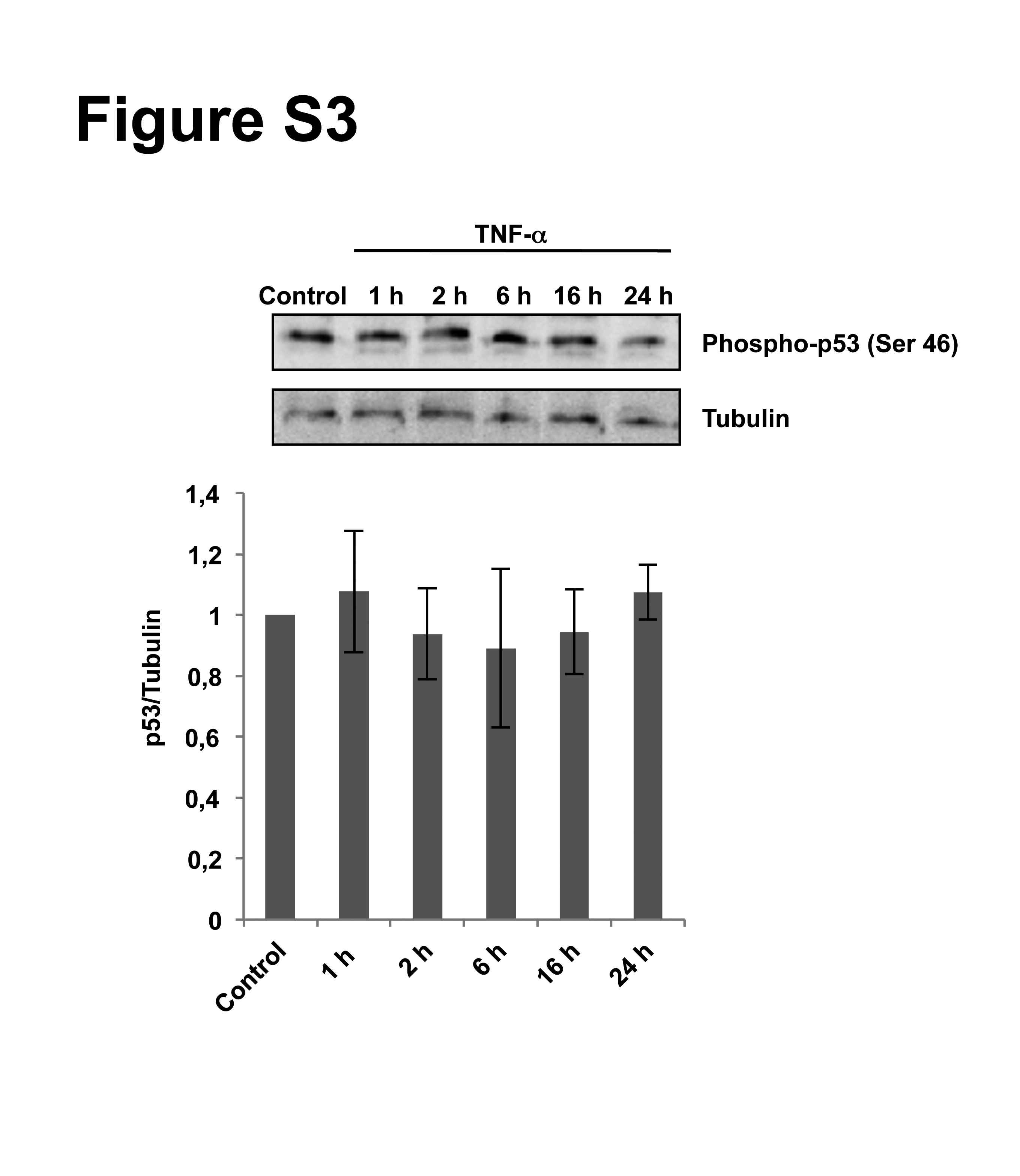

Supplement: Figure S3 — P53 is not involved in TNF-α-mediated cell death. p53-PSer46 protein levels were determined by Western blot analysis. Cell lysates from untreated or stimulated cells for the indicated times were separated by SDS-PAGE on 10% acrylamide gel, blotted, and incubated with antibodies against p53-PSer46. (TIF) [file pone.0016100.s003.tif]
